# New Perspectives on the Interactions between Adsorption and Degradation of Organic Micropollutants in Granular Activated Carbon Filters

**DOI:** 10.1021/acs.est.4c00815

**Published:** 2024-06-18

**Authors:** Alexander Betsholtz, Per Falås, Ola Svahn, Michael Cimbritz, Åsa Davidsson

**Affiliations:** † Department of Process and Life Science Engineering. Division of Chemical Engineering, Lund University, Lund 221 00, Sweden; ‡ School of Education and Environment, Division of Natural Sciences, Kristianstad University, Kristianstad 291 88, Sweden

**Keywords:** biological degradation, pharmaceuticals, retention

## Abstract

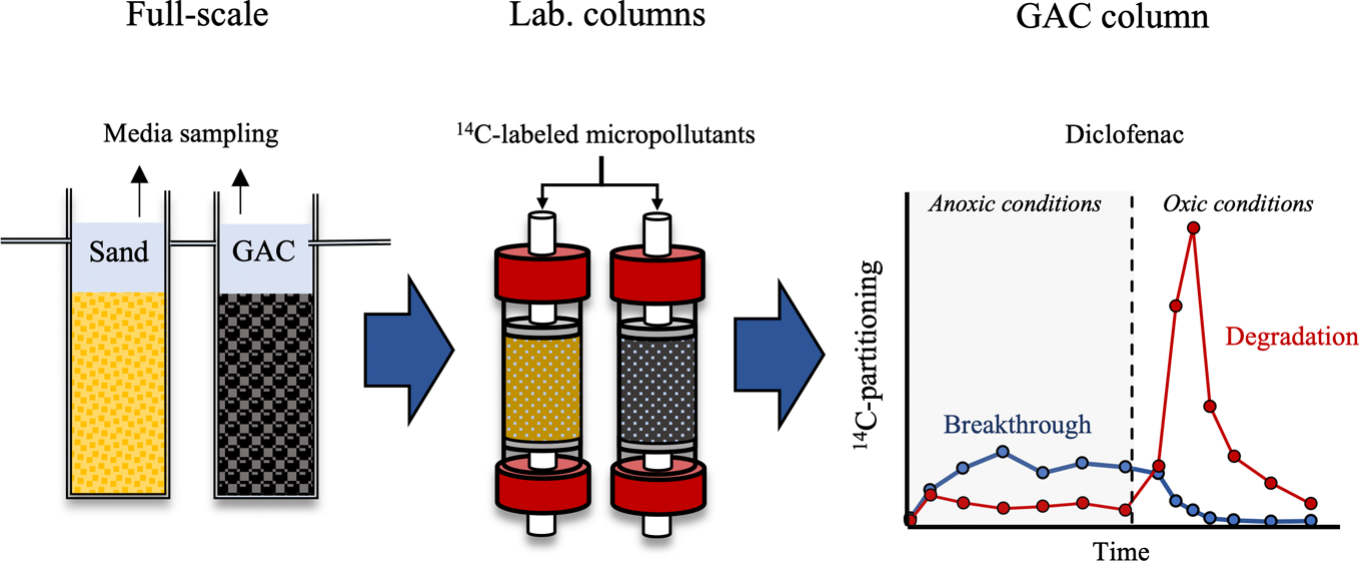

The removal of organic micropollutants in granular activated carbon (GAC) filters can be attributed to adsorption and biological degradation. These two processes can interact with each other or proceed independently. To illustrate the differences in their interaction, three ^14^C-labeled organic micropollutants with varying potentials for adsorption and biodegradation were selected to study their adsorption and biodegradation in columns with adsorbing (GAC) and non-adsorbing (sand) filter media. Using ^14^CO_2_ formation as a marker for biodegradation, we demonstrated that the biodegradation of poorly adsorbing *N*-nitrosodimethylamine (NDMA) was more sensitive to changes in the empty bed contact time (EBCT) compared with that of moderately adsorbing diclofenac. Further, diclofenac that had adsorbed under anoxic conditions could be degraded when molecular oxygen became available, and substantial biodegradation (≥60%) of diclofenac could be achieved with a 15 min EBCT in the GAC filter. These findings suggest that the retention of micropollutants in GAC filters, by prolonging the micropollutant residence time through adsorption, can enable longer time periods for degradations than what the hydraulic retention time would allow for. For the biologically recalcitrant compound carbamazepine, differences in breakthrough between the ^14^C-labeled and nonradiolabeled compounds revealed a substantial retention via successive adsorption–desorption, which could pose a potential challenge in the interpretation of GAC filter performance.

## Abstract 2

Adsorption in GAC filters decouples micropollutant degradation time from hydraulic residence time, which could facilitate the degradation of adsorbable compounds.

## 1 Introduction

Granular activated carbon (GAC) filters are becoming an increasingly applied option for the full-scale abatement of organic micropollutants in municipal wastewater.^1−5^ Empty bed contact time (EBCT) has been proposed as a key operating parameter to allow sufficient time for the adsorption of micropollutants in GAC filters^6^ but could also influence the biodegradation of micropollutants by GAC biofilms. EBCTs typically range from 10 to 45 min,^7^ which is considerably lower than the hydraulic retention time (HRT) for most biological wastewater treatment processes (4–20 h).^8^ Thus, the EBCT appears to be insufficient to obtain any significant biodegradation of organic micropollutants in GAC filters.^9^

Nevertheless, the sustained removal of certain biodegradable micropollutants at high numbers of treated bed volumes indicates the importance of biodegradation as an important long-term removal mechanism for these micropollutants.^10−12^ These observations suggest that adsorption may be central to achieving high biodegradation in GAC filters. Yet, the potential interactions between the adsorption and biodegradation of micropollutants in GAC filters remain poorly understood due to the challenges in separating biodegradation from adsorption.

To this end, the individual contributions of biodegradation and adsorption have primarily been estimated indirectly through the observed difference between a biologically active GAC filter and a sterilized GAC filter.^13−20^ In wastewater, the use of this approach has indicated a positive contribution of GAC biofilms to the removal of several biodegradable micropollutants, such as diclofenac and sulfamethoxazole.^13,15,20^ However, comparing biologically active and sterilized filters has inherent limitations. The sterilization methods that are applied might be unable to inhibit biological activity—completely or selectively—without affecting adsorption conditions.^21^ Further, the observed differences between a biologically active and sterilized filter cannot be used to discern whether the mechanism by which micropollutant removal increases is attributed to direct biodegradation of the micropollutant or to greater adsorption from the liberation of adsorption sites through the biodegradation of competing dissolved organic matter.

The use of ^14^C-labeled organic micropollutants constitutes a direct method for separating biodegradation from adsorption when the ^14^C-labeled moiety is mineralized to ^14^CO_2_, which adsorbs poorly to activated carbon and can be extracted with CO_2_ traps.^22,23^ By measuring the radioactive decay (ß-particles) from disintegration of ^14^C, the labeled carbon(s) can be tracked between different liquid phases by liquid scintillation counting. In particular, measurements of ^14^CO_2_ formation with CO_2_ traps can be used to quantify the extent of degradation that occurs via mineralization (^14^CO_2_ formation) of the labeled moiety but no other moieties. This ^14^C-approach has been used to study the biodegradation and bioregeneration of selected drinking water contaminants in small-scale GAC columns^24,25^ but has not been applied in column-based experiments for treating organic micropollutants in wastewater.

The objective of this study was to examine the interactions between adsorption and biodegradation with regard to the removal of organic micropollutants in the GAC filters. We selected 3 micropollutants with varying adsorption affinities to activated carbon and differing potential for biodegradation: diclofenac, a biodegradable and adsorbable compound; *N*-nitrosodimethylamine (NDMA), a biodegradable and weakly adsorbable compound; carbamazepine, a nonbiodegradable and adsorbable compound. We hypothesized that the biodegradation of adsorbable and biodegradable compounds (represented by diclofenac) is less sensitive to changes in EBCT than that of non-adsorbable and biodegradable compounds (represented by NDMA). To test this hypothesis, we studied ^14^C-labeled and nonradiolabeled micropollutants in laboratory-scale column experiments with adsorptive GAC media and non-adsorptive sand media that had been retrieved from a full-scale treatment process.

## 2 Materials and Methods

**Figure 1:**
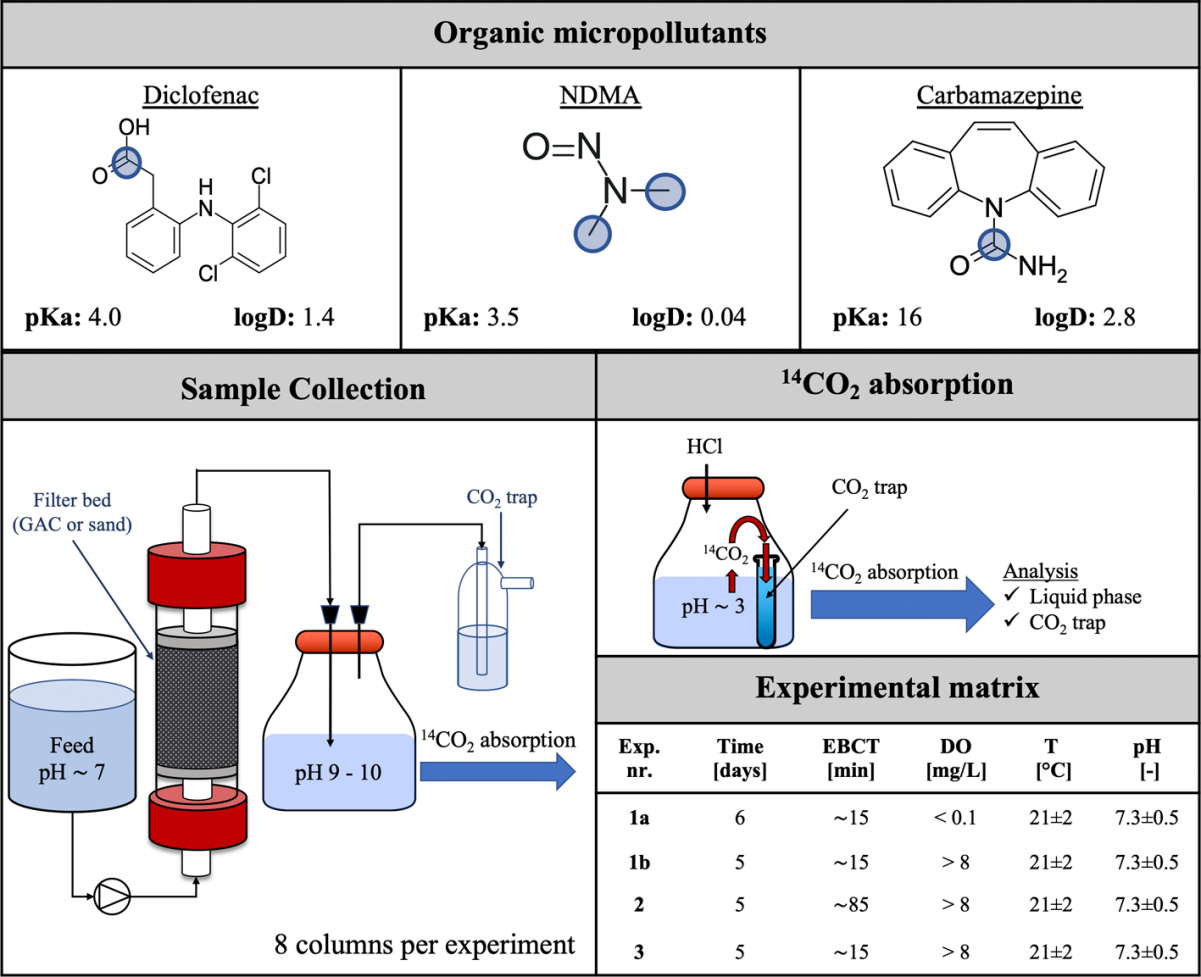
Overview of selected micropollutants, experimental setup, and operational parameters. The ^14^C-labeled moieties are indicated by blue circles. Chemical structures, p*K*_a_ values, and octanol–water partitioning coefficients at pH 7 (logD) were obtained using Chemicalize, 2023.04 (ChemAxon, https://chemicalize.com/).

To examine the biodegradation of previously adsorbed micropollutants and the effect of EBCT, 3 separate experiments were performed with GAC and sand filter media from Degeberga WWTP, collected at ∼25,000 bed volumes (BVs). Each experiment comprised 8 columns that were operated for up to 1000 BVs to study the adsorption and biodegradation of the selected compounds. An overview of these micropollutants, their ^14^C labeling, and the experimental setup is shown in Figure 1 and detailed in Sections 2.1–2.5.

### 2.1 Micropollutant Selection

Three ^14^C-labeled organic micropollutants with different potentials for biodegradation and adsorptive properties were selected (Figure 1): carbamazepine [carbonyl-^14^C], diclofenac [carboxyl-^14^C], and *N*-nitrosodimethylamine (NDMA, [methyl-^14^C]) (all from Hartmann Analytics, Germany). Carbamazepine is a nonbiodegradable^26^ anticonvulsant drug with high adsorptive potential to activated carbon.^27^ Diclofenac is a nonsteroidal anti-inflammatory drug that undergoes low removal in biological wastewater treatment^28^ but is potentially biodegradable in GAC filters,^10^ with medium adsorptive affinity to activated carbon.^29^ NDMA is a small, polar, and biodegradable carcinogen,^30^ with low adsorptive affinity to activated carbon.^31,32^ The ^14^C-labeled forms of these micropollutants are hereafter referred to as ^14^C-diclofenac, ^14^C-carbamazepine, and ^14^C-NDMA, respectively. All ^14^C positioning was selected based on commercial availability, and the radiochemical purities were >98%. Nonradiolabeled micropollutants of analytical grade were also used for the experiments with subsequent UPLC-MS/MS analysis.

### 2.2 Experimental Design

Three experiments were performed in the dark at 21 ± 2 °C and a pH of 7.3 ± 0.5 but under different redox conditions and EBCTs (Figure 1). Experiment 1 was used to study whether previously adsorbed micropollutants could be degraded and was divided into two phases: 6 days of anoxic conditions (DO < 0.1 mg/L) with feeding of ^14^C-labeled micropollutants (experiment 1a), followed by 5 days of oxic conditions (DO > 8 mg/L) without feeding of ^14^C-labeled micropollutants (experiment 1b). The objective of the anoxic conditions in experiment 1a was to limit biological degradation, thus accumulating micropollutants in the GAC column via adsorption. The EBCT was ∼15 min in both phases. In the anoxic phase, low-oxygen conditions were established by sparging the feed with nitrogen gas overnight and performing the experiment in a glovebox with continuous flow of nitrogen gas. To reduce the risk of concentration-induced desorption of ^14^C in the oxic phase (experiment 1b) due to the termination of ^14^C in the feed, nonradiolabeled micropollutants were added individually to the feed at concentrations that corresponded to the ^14^C added during the anoxic phase (experiment 1a).

Experiments 2 and 3 were designed to study the effect of the empty bed contact time on micropollutant adsorption and biodegradation (Figure 1). Experiment 2 was conducted with long EBCT (85 min), and experiment 3 was conducted with short EBCT (15 min). Both experiments were performed over 5 days under oxic conditions (DO > 8 mg/L).

### 2.3 Experimental Setup

#### 2.3.1 Full-Scale Treatment at Degeberga WWTP and Media for Column Experiments

GAC and sand were retrieved from the top of two full-scale filters at Degeberga WWTP, Sweden, on three separate occasions (Figure S1 and Table S1). GAC from the top of the filter bed was used to best resemble long-term operation in terms of adsorbed micropollutants^33^ and biofilm development.^34^ The sand and GAC filters are operated in series, downstream of an activated sludge process with nitrification and partial denitrification, followed by postprecipitation and final clarification. The sand filter, the first to be established in Sweden, has been in operation since 1975 and has a current hydraulic surface loading of 1 m/h, a bed depth of 1.4 m, a mean EBCT of 87 min, and high dissolved oxygen (DO > 8 mg/L) in the influent.

The GAC filters, installed in April 2020, constitute the first full-scale GAC treatment of municipal wastewater in Sweden, and they were scaled up from a previous pilot-scale study—the FRAM Project^22,35^—with sequential sand and GAC filters that have been operated for over 40,000 BVs. The GAC filters consist of open basins that are aerated by overfall, ensuring high dissolved oxygen concentrations (DO > 8 mg/L) in the influent. The sampled GAC filter is operated with the same bituminous coal-based activated carbon as in the FRAM Project (Aquasorb 5000, 8′30 mesh, 0.6–2.4 mm, Jacobi), with a specific Brunauer–Emmett–Teller (BET) surface area of 1200 m^2^/g. The GAC used was selected based on adsorption studies with 9 different commercial GAC types using an LC-UV-based technique,^36^ where it showed the highest adsorption ability, highest removal of negatively charged compounds, and fast adsorption kinetics. The filter has a bed depth of 1 m and is operated with a mean EBCT of 45 min and a hydraulic surface loading of 1.3 m/h. At the time of media retrieval for the column experiments, the GAC filter had been running for over 2.5 years and over 25,000 BVs without any backwashing events.

In the full-scale filters, the removal of carbamazepine was 30–50% in the GAC filter and ±5% in the sand filter at the time for this investigation (Table S1). For diclofenac, on the other hand, the removal was 80–90% in the GAC filter and 15–40% in the sand filter. Detailed information on the GAC filter performance and the removal of the 24 monitored organic micropollutants can be found elsewhere^2^ (please contact Ola Svahn (ola.svahn@hkr.se) for details regarding the FRAM Project and the advanced treatment in Degeberga).

After media retrieval, the GAC and sand were stored at 8 °C for <72 h before the start of the experiment. Before use, suspended solids that might have originated from previous treatment steps were removed from the GAC and sand media through careful, repeat washes with tap water. In addition, medium size fractions >1.5 mm were eliminated by carefully passing the media through a 1.5 mm sieve under water. The effective size of the resulting GAC and sand was determined via sieving per Swedish standards (SS-EN ISO 17892-4:2016). The resulting ratios of the inner diameter (16 mm) of the column to the mean particle diameter ranged from 14.8 to 15.4 for the GAC media and from 17.4 to 19.3 for the sand media (Table S2), which exceed the minimum ratios (5–10) that have been recommended to avoid wall effects.^37,38^

#### 2.3.2 Column Setup

Gas-tight glass columns (length of 200 mm, inner diameter of 16 mm; Cytiva) with adapters for adjustable bed heights were filled with media (GAC or sand) to a volume of 10 cm^3^, corresponding to a bed height of ∼50 mm and dry weights of ∼3 g GAC and ∼15 g sand (Table S3). The feed consisted of biologically and chemically treated wastewater that was retrieved downstream of the sand filter at the Degeberga WWTP and spiked with radiolabeled or nonradiolabeled micropollutants. Before use, the water was filtered (0.45 μm cellulose nitrate, Whatman), aerated (dissolved oxygen, DO > 8 mg/L), and adjusted to pH 7 with 1 M NaOH or HCl.

Eight columns were used in each experiment and divided into 4 pairs, each of which consisted of one GAC filter and one sand filter. Three pairs of columns were fed with ^14^C-labeled micropollutants (one micropollutant per column pair) at ^14^C activities of 0.1 μCi/L, corresponding to concentrations of 100–1100 ng/L (Table S4). The last pair was used to study the nonradiolabeled compounds and was spiked with the same amounts of the corresponding nonradiolabeled micropollutants as with the ^14^C-labeled micropollutants, and the resulting concentrations were thus the sum of the spiked and background levels (Table S4). The last pair was also used to follow the concentrations of standard parameters.

#### 2.3.3 Column Operation

Before the start of the experiments, the filters were adapted to the experimental conditions (EBCT, T, DO) by feeding them wastewater without spiking of ^14^C or nonradiolabeled micropollutants for 12–24 h before ^14^C-labeled micropollutants were added to the feed.

The effluent from each filter was collected over time into several sets of sealed glass bottles through a hypodermic needle (Figure 1). A secondary needle was used to purge excess air through a CO_2_ trap (25 mL, 1 M NaOH) to monitor ^14^CO_2_ loss from the bottle during sample collection. To avoid ^14^CO_2_ loss, the bottles contained small, predetermined amounts of 1 M NaOH to achieve a final pH of 9–10, at which dissolved inorganic ^14^C exists primarily as ^14^CO_3_^2–^ and H^14^CO_3_^–^. On complete retrieval of the sample for each interval, a glass-tube CO_2_ trap (30 mL, Ø 1.5 cm) that contained 20 mL of 1 M NaOH was installed carefully in the glass bottle, after which predetermined amounts of 1 M HCl were added to lower the pH to 3 ± 0.2, favoring CO_2_ release. The bottles were then sealed immediately and incubated for 40–48 h in the dark at 20 °C and 130 rpm to allow complete absorption of CO_2_ by the trap. After incubation, the samples were retrieved from the trap and liquid phase and transferred to 10 mL Falcon tubes for subsequent analysis of ^14^C activity. At the end of the experiment, all remaining GAC and sand from each column were frozen immediately for a later analysis of adsorbed ^14^C by combustion and subsequent CO_2_ trapping.

### 2.4 Analysis

#### 2.4.1 ^14^C Measurements

^14^C activity was measured in disintegrations per minute (DPM) by liquid scintillation counting on a Tricarb 4910 TR (PerkinElmer) using a scintillation cocktail (Ultima Gold, PerkinElmer). Quenching was accounted for using transformed Spectral Index of the External Standard values.^39^ For the column experiments, 0.6 mL of the 1 M NaOH traps was added to a final volume of 4 mL in 6 mL polypropylene vials. For the liquid wastewater samples, 4 mL was added to a total of 16 mL in 20 mL polypropylene vials. For the combustion experiments, 0.6 mL of the 4 M NaOH traps was added to a total of 16 mL in 20 mL polypropylene vials. The background radiation (*n* = 3) in NaOH and wastewater was subtracted from the value of each measurement.

To measure ^14^C in the solid phase, portions of the remaining GAC (1.00 ± 0.05 g wet weight) or sand media (2.00 ± 0.05 g wet weight) were combusted in triplicate in a Pyrolyzer^genIII^ (Raddec International) that was equipped with downstream CO_2_ traps (2 traps in series with 25 mL of 4 M NaOH each). Dry weight:wet weight ratios were determined, based on triplicate measurements, before and after drying at 105 °C. The combustion program was run at an air flow rate of ∼0.1 L/min, with stepwise heating and dwelling to 900 °C over ∼5 h and the catalyst zone maintained at 900 °C during the entire combustion run, as detailed in Table S5. The combustion method had a ^14^C recovery of >90% for ^14^C-diclofenac and ^14^C-carbamazepine and >80% for ^14^C-NDMA for adsorbed compounds to virgin GAC.

#### 2.4.2 Micropollutant Analysis

Wastewater samples (50 mL) that contained nonradiolabeled micropollutants were concentrated by solid-phase extraction (SPE) and analyzed by ultraperformance liquid chromatography (UPLC), coupled with tandem mass spectroscopy (MS/MS), as detailed elsewhere.^40,41^ Briefly, the samples were extracted on SPE columns (Oasis HLB 200 mg). After being dried, eluted, and evaporated, the samples were reconstituted and injected (1 μL) into the UPLC MS/MS instrument (Waters Acquity UPLC H-Class, Xevo TQS Waters Micromass, Manchester, UK). Limits of quantification (LOQs) and relative standard deviations (RSDs) for diclofenac and carbamazepine are provided in Table S6. Nonradiolabeled NDMA was not measured in the experiments.

#### 2.4.3 Other Parameters

Wastewater parameters were measured after filtration (0.45 μm cellulose nitrate, Whatman). Dissolved organic carbon (DOC) was analyzed on a Shimadzu TOC-L (Shimadzu Scientific Instruments, Columbia, MD). Ultraviolet absorption at 254 nm, UVA_254_, was determined on a Dr6000 spectrophotometer (Hach) in a 5 cm quartz cuvette. Ammonium (NH_4_^+^-N), nitrite (NO_3_^–^-N), and nitrite (NO_2_^–^-N) were measured by ion chromatography (ECO IC, Metrohm, Switzerland).

### 2.5 Data Presentation

The results from the column experiments are presented as the mass flow of ^14^C per unit time or accumulated fractions (eq 1). Accumulated fractions were used to graphically visualize data even after the addition of ^14^C-labeled micropollutants to the influent had been terminated (Experiment 1b) and was used to present the data from all experiments.
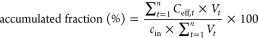
1where *t* is the sampling time point, *n* is the time point at which the accumulated fraction is calculated, *c*_in_ is the influent concentration, *c*_eff,*t*_ is the effluent concentration between time point *t* and *t* – 1, and *V*_*t*_ is the volume collected between time point *t* and *t* – 1.

## 3 Results and Discussion

A total of 24 columns with GAC and sand filter media were run over 5 or 11 days to evaluate the biodegradation and adsorption of diclofenac, NDMA, and carbamazepine under various redox conditions and EBCTs. The experimental conditions and influent/effluent concentrations of the standard wastewater parameters are provided in the Supporting Information for the initial adaptation period (Table S7) and the corresponding analysis of diclofenac and carbamazepine in the nonradiolabeled columns (Table S8). Low removal of DOC (<16%) and UVA_254_ values were observed, as expected, based on the high number of treated BVs (>25,000 BVs) in the full-scale GAC filter. The flow through the columns was generally stable (Figure S2), with similar contact times, in the 8 columns in the experiments with anoxic/oxic conditions (16.2 ± 0.7 min) and oxic conditions with long EBCT (85 ± 2.6 min) and short EBCT (15.4 ± 0.5 min).

### 3.1 Biodegradation of Previously Adsorbed Micropollutants

**Figure 2:**
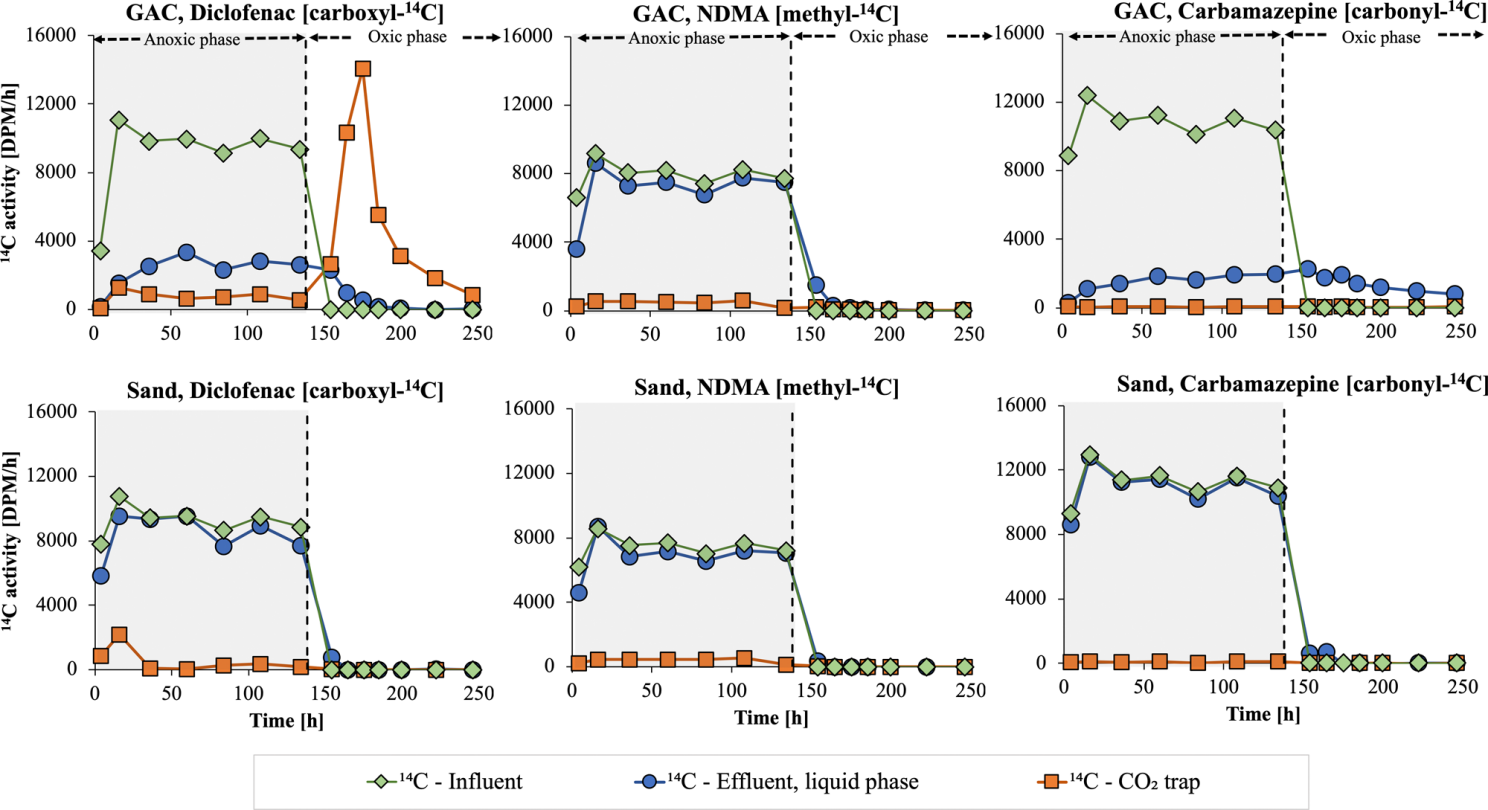
Flux of ^14^C in the influent (green diamonds) and effluent (liquid phase; blue circles) and to the CO_2_ trap (orange squares) of the columns during the anoxic/oxic experiment at an EBCT of 15 min. After the anoxic phase (DO < 0.1 mg/L) (shaded), oxic (DO > 8 mg/L) conditions were introduced but without the addition of ^14^C.

To determine whether micropollutants could be adsorbed and degraded at a later stage, ^14^C-labeled micropollutants were allowed to adsorb during an initial anoxic phase (Experiment 1a), with a subsequent oxic phase, without ^14^C being fed (Experiment 1b). The EBCT was ∼15 min in both phases. The anoxic conditions in the initial phase were confirmed through stable NH_4_^+^-N concentrations compared with those in the oxic phase (Tables S7 and S8). Limited ^14^CO_2_ formation was observed in the anoxic phase for ^14^C-diclofenac (<10%), ^14^C-NDMA (<5%), and ^14^C-carbamazepine (<1%) in the GAC and sand columns (Figure 2). The degradation of diclofenac has been reported to be limited under anoxic conditions.^42−44^ Anoxic removal of NDMA has been observed at high residence times (days) in soil,^45^ and dealkylation of the ^14^C-labeled methyl carbon has been confirmed through the formation of ^14^CO_2_.^46^

For GAC, ^14^CO_2_ formation from ^14^C-diclofenac increased in the oxic phase (Figure 2), which must have originated from previously adsorbed ^14^C and might have consisted of both ^14^C-diclofenac and ^14^C-labeled products that resulted from anoxic transformation. Transformation products that are generated from the cleavage of the labeled carboxylic carbon in diclofenac have also been reported for aerobic biofilms. However, among the primary transformation pathways reported for diclofenac (hydroxylation, decarboxylation, and amidation), only decarboxylation directly leads to a cleavage of the ^14^C-labeled carboxylic carbon.^47^ Further transformation of the other primary transformation products may, however, also lead to cleavage of the labeled carbon.

The rate of ^14^CO_2_ formation rose immediately after the introduction of dissolved oxygen, with its peak exceeding the inflow of ^14^C-diclofenac per time unit in the anoxic phase. These results suggest that previously adsorbed diclofenac can be degraded under oxic conditions and that the biofilm can attain biodegradation rates that are sufficiently high to remove substantial amounts of diclofenac. For the sand column, however, negligible ^14^CO_2_ formation was observed in the oxic phase, as expected, based on the limited adsorption to sand in the anoxic phase.

The adsorption of ^14^C-NDMA was low (<10%) in the GAC and sand columns during the anoxic phase, as expected, based on the polar nature of NDMA and its low adsorptive affinity to activated carbon.^31,32^ Therefore, the potential for the biodegradation of previously adsorbed NDMA in the oxic phase was limited. In contrast, ^14^C-carbamazepine partially adsorbed to the GAC column during the anoxic phase, but its biological recalcitrance^28^ prevented its subsequent degradation (or potential ^14^CO_2_ formation) under oxic conditions.

**Figure 3:**
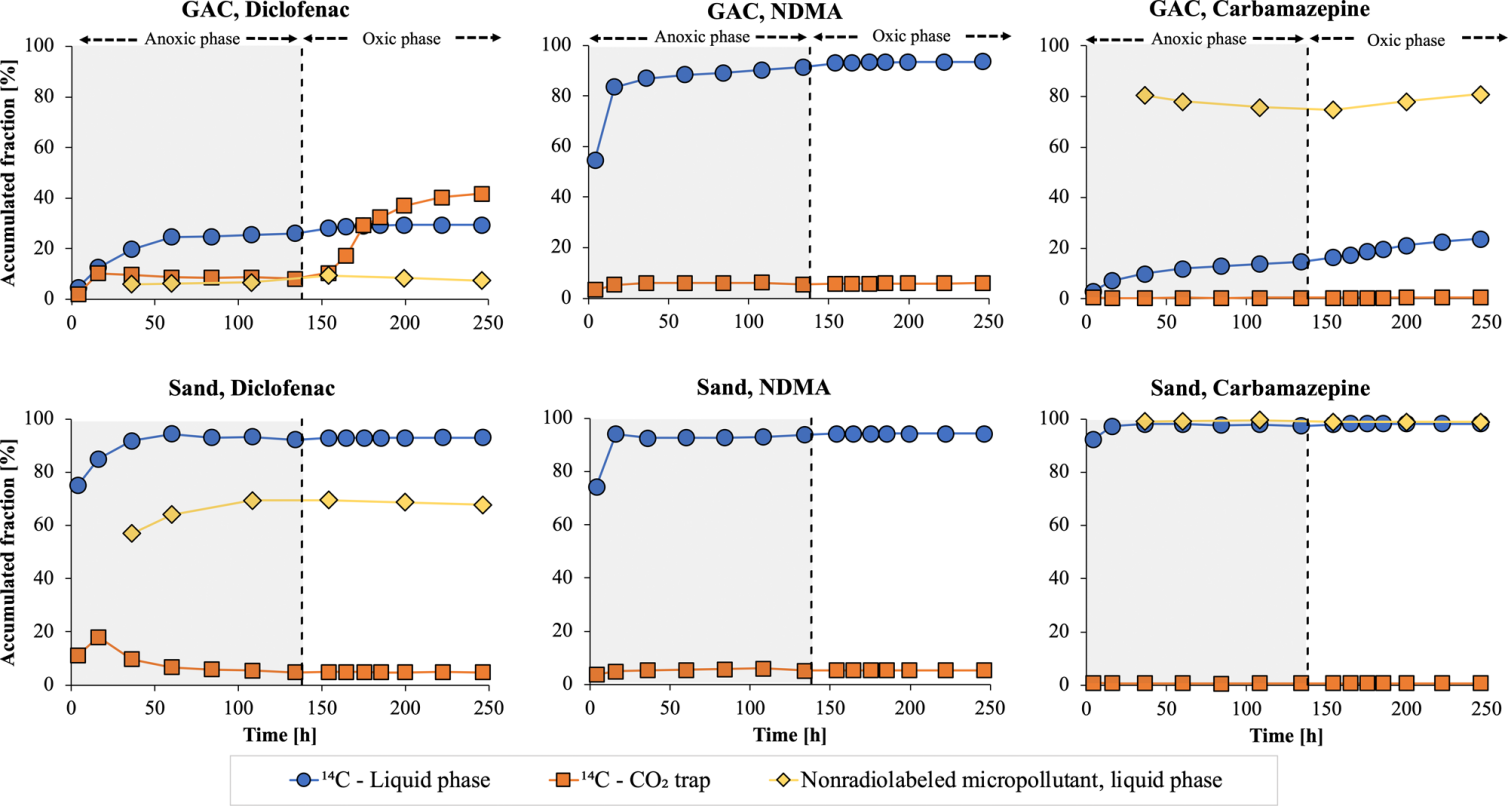
Accumulated fractions of ^14^C in the liquid phase (blue rings) and the CO_2_ trap (orange squares) and nonradiolabeled micropollutant (yellow diamonds) in the anoxic–oxic experiment (experiment 1). Please note that the data for the nonradiolabeled and ^14^C-labeled compounds were obtained in separate columns operated in parallel.

Figure 3 shows the accumulated fractions (eq 1) of ^14^C in the effluent liquid phase and the CO_2_ trap and those of the nonradiolabeled compounds (diclofenac and carbamazepine) in the effluent liquid phase. Low accumulated fractions in the effluent liquid phases can be interpreted as high removal over the column (or low breakthrough), whereas high accumulated fractions of ^14^CO_2_ can be interpreted as high biological degradation of the ^14^C-labeled moieties. The removal of nonradiolabeled diclofenac and carbamazepine was stable over time but differed between compounds and columns. The removal of ^14^C from ^14^C-diclofenac was lower than the removal of nonradiolabeled diclofenac in the GAC and sand columns, suggesting that biodegradation of diclofenac occurs in the anoxic phase and that some of the ^14^C in the liquid phase effluent (∼25% for the sand filter and ∼20% for the GAC filter) consisted of ^14^C-labeled transformation products. These observations contrast the results of previous studies on anoxic transformation of diclofenac in sludge^42,43^ and moving bed biofilm reactors.^44,48^ Anoxic transformation of diclofenac has however been indicated during managed aquifer recharge,^49^ but there are no such data for rapid sand filtration.

For ^14^C-NDMA, the level of ^14^CO_2_ formation was ∼5% in the anoxic phase for both the sand and GAC columns. However, the low adsorption of ^14^C-NDMA in the anoxic phase for the sand column (∼5%) and the GAC column (∼10%) limited the degradation in the oxic phase when the addition of ^14^C-NDMA had been terminated.

Limited removal of carbamazepine (<5%) was observed in the sand column based on the analysis of both the ^14^C-labeled and nonradiolabaled compounds. However, a clear difference (50–70%) was observed between the removal of ^14^C-labeled and nonradiolabeled carbamazepine in the GAC column. For ^14^C-carbamazepine, the breakthrough increased slowly but steadily, even after the addition of ^14^C-carbamazepine had been terminated in the oxic phase. These results show that ^14^C-carbamazepine was substantially retained on its travel through the GAC column via successive adsorption–desorption and that the adsorption was at least partially reversible. The higher breakthrough of nonradiolabeled carbamazepine can be explained by the desorption of carbamazepine that had been adsorbed prior to the experiments during the operation of the full-scale filter (∼25,000 BVs). Whereas nonradiolabeled carbamazepine was preloaded throughout the GAC bed and could have desorbed from all areas of the column, newly introduced ^14^C-carbamazepine had to travel through the entire GAC bed to reach the effluent. Although it may appear as a large portion of carbamazepine that passed through the bed without being adsorbed, our results with ^14^C-labeled carbamazepine show that the compound is substantially retained on its passage through the GAC bed via adsorption–desorption interactions.

After these experiments, ^14^C mass balances were established via combustion of the GAC and sand filter media and subsequent ^14^CO_2_ trapping, resulting in >75% recovery of the ^14^C for all compounds (Figure S3). Higher uncertainties were generally observed with higher degrees of biological mineralization (^14^CO_2_ formation); one explanation is that it is easier to recover stable and recalcitrant compounds in one or two phases compared to biodegradable compounds with potentially volatile transformation products in three phases.

### 3.2 Influence of EBCT

**Figure 4:**
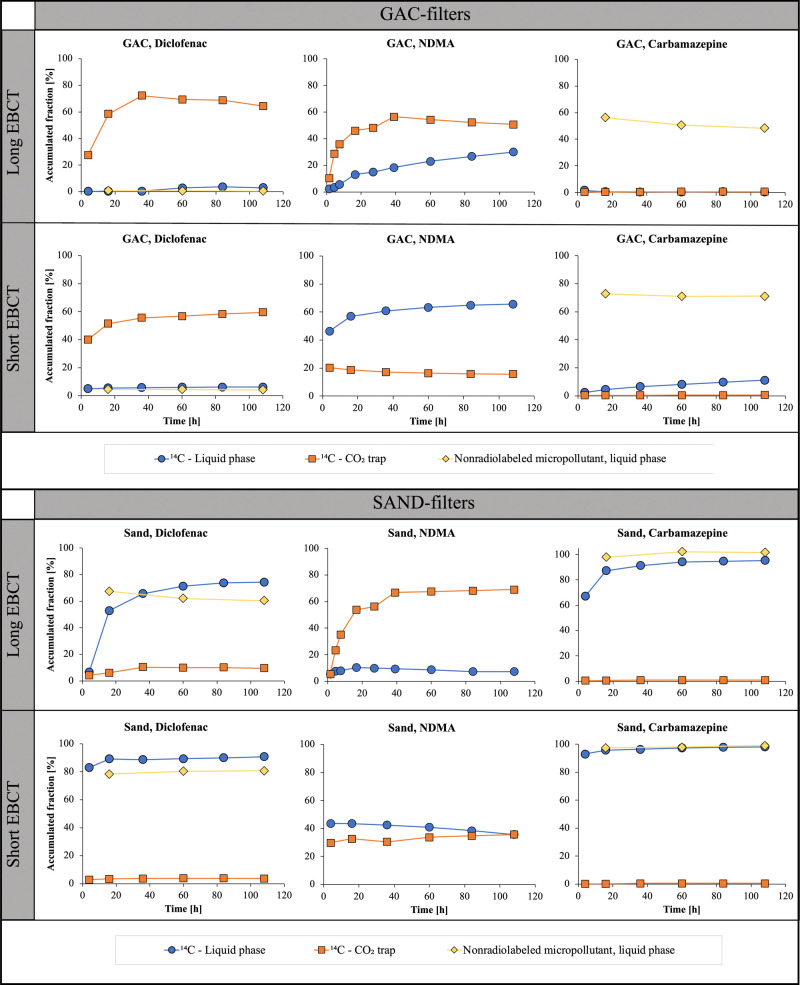
Accumulated fractions of ^14^C in the liquid phase (blue rings) and CO_2_ trap (orange squares) in the columns with ^14^C-labeled micropollutants and nonradiolabeled micropollutant (yellow diamonds) in the experiments with short and long EBCTs (experiments 2 and 3). Please note that the data for the nonradiolabeled and ^14^C-labeled compounds were obtained in separate columns operated in parallel.

To further investigate the influence of the EBCT on micropollutant adsorption and biodegradation, we performed column experiments at short (∼15 min; experiment 2) and long (∼85 min; experiment 3) EBCTs with GAC and sand columns (Figure 4 and Figure S3) without an initial anoxic phase. ^14^C-diclofenac and ^14^C-NDMA were partially degraded at their respective ^14^C-labeled moieties, as evidenced by the ^14^CO_2_ formation in both columns; further, the extent of ^14^CO_2_ formation was higher than for the anoxic phase in the previous experiment (Figure 3).

There was substantial biodegradation of ^14^C-diclofenac in the GAC column at both the short and long EBCTs (∼60 and ∼70% ^14^CO_2_ formation at EBCTs of 15 and 85 min, respectively), accompanied by low breakthrough (0–10%) of liquid-phase ^14^C. Low breakthrough (<5%) was also observed for nonradiolabeled diclofenac compared with the full-scale GAC filter during GAC sampling (10–20% at an EBCT of ∼45 min; Table S1). Increasing the EBCT from 15 to 85 min only resulted in a slightly higher ^14^CO_2_ formation, a slightly lower ^14^C breakthrough in the liquid phase, and a lower breakthrough of nonradiolabeled diclofenac.

The formation of ^14^CO_2_ in the GAC columns (∼60% at a 15 min EBCT) is high compared with the values in the literature for nonradiolabeled diclofenac removal in biofilm systems, in which hydraulic retention times of 4–16 h have been required for ∼80% removal in high-performing systems.^47,50^ In addition, the observed formation of ^14^CO_2_ from diclofenac is higher than expected from previous comparisons between biologically active and sterilized GAC;^13,51^ biodegradation, however, might have been masked by high adsorption in the sterilized column.^20^ Overall, our results support previous indications that biodegradation of diclofenac underlies the sustained removal of diclofenac in full-scale GAC filters at higher bed volumes (>25,000) and short EBCTs (16–25 min).^10,52^

In the sand columns, ^14^CO_2_ formation from ^14^C-diclofenac (∼5% at a 15 min EBCT and ∼10% at an 85 min EBCT) was much lower than for GAC. Lower effluent fractions were observed for nonradiolabeled diclofenac compared with liquid-phase ^14^C (similar to the anoxic experiment), suggesting that biodegradation also proceeded via pathways that resulted in the formation of ^14^C-labeled transformation products, such as hydroxy-diclofenac.^10,47^ The removal of nonradiolabeled diclofenac in the sand columns with similar biofilm content (expressed as volatile solids, Table S3) ranged from ∼20% (15 min EBCT) to ∼40% (85 min EBCT), which is comparable with the values in the literature, ∼ 20% at a 120 min EBCT^11^ and 20–30% at an EBCT of 8–16 min,^53^ and consistent with what has been observed for the full-scale sand filter at Degeberga WWTP (15–40% in Table S1 and a 3 year average of 35%^2^).

In contrast to ^14^C-diclofenac, more ^14^CO_2_ was formed from ^14^C-NDMA in the sand (35%, short EBCT; 70%, long EBCT) versus GAC columns (15%, short EBCT; 50%, long EBCT). Aerobic transformation pathways of NDMA are well documented in mammalian cells^54^ and have been proposed to proceed via similar pathways in bacteria expressing specific monooxygenase enzymes.^55^ The transformation is expected to occur either through an initial hydroxylation of one of the methyl groups^55^ or via an initial oxidation of the nitroso group into a nitro group, followed by a hydroxylation of one of the methyl groups.^56^ Subsequent demethylation of the hydroxylated intermediates has been reported to result in formaldehyde formation, where further transformation is expected to result in mineralization of one of the labeled carbons. Aerobic mineralization of the ^14^C-labeled carbon in ^14^C-NDMA has also been demonstrated for aqueous and soil systems.^57^

Similar removal of nonradiolabeled NDMA (50–90%) has previously been reported for sand and GAC filters.^30^ By increasing the EBCT from 15 to 85 min, the fraction of ^14^CO_2_ formation from ^14^C-NDMA rose from 35 to 70% for sand and from 20 to 50% for GAC (Figure 4). These results suggest that GAC filter media do not necessarily have higher overall biodegradative capacity for organic micropollutants than sand filter media. This could to some extent also limit the possibilities for predicting the biodegradation capacity of organic micropollutants in GAC filters based on degradation data from other biological wastewater treatment processes. For the GAC columns, the liquid-phase ^14^C fractions in the effluent from ^14^C-NDMA were higher than for diclofenac (∼60% at a 15 min EBCT and ∼25% at an 85 min EBCT), illustrating that degradation, rather than adsorption, governs the removal of NDMA. Overall, the removal of poorly adsorbing NDMA was more sensitive to changes in EBCT compared with well-adsorbing diclofenac. These results strengthen the theory that by prolonging the time for biodegradation for adsorbable and biodegradable substances in GAC filters, adsorption can improve the conditions for biodegradation.

A longer EBCT decreased the breakthrough of nonradiolabeled carbamazepine in GAC columns from ∼70 to ∼50%. In contrast, the breakthrough of ^14^C-carbamazepine in the GAC columns was negligible at a long EBCT but increased slowly but steadily over time at the short EBCT. Decreased removal and earlier breakthrough are expected at shorter EBCTs due to mass transfer limitations of the adsorption process at EBCTs <20 min^6^ but also to the higher number of BVs passing the columns (∼480 BV at the short EBCT and ∼85 BV at the long EBCT) in the experiments with ^14^C-labeled carbamazepine. As shown in the previous anoxic–oxic experiment, the large differences between the fractions of liquid-phase ^14^C and nonradiolabeled carbamazepine highlight the slow transport of carbamazepine through the columns via continuous adsorption–desorption.

### 3.3 Implications

The observed degradation of previously adsorbed diclofenac illustrates that the retention in GAC filters prolongs the time available for biodegradation, thereby decoupling the biodegradation time from the hydraulic residence time. This retention increases the micropollutant concentration locally (adsorbed + liquid phase), which may allow for higher micropollutant biodegradation rates in terms of mass^58,59^ and/or increase micropollutant availability, potentially promoting the selection of microorganisms targeting their removal. Thus, by prolonging the micropollutant residence time through adsorption in GAC filters, it may be possible to achieve high removal of certain micropollutants at hydraulic residence times that are normally considered insufficient for substantial biodegradation in biological wastewater treatment processes.^28^

Still, not all potentially biodegradable micropollutants may be available for biotransformation in GAC filters due to the expected adsorption in micropores and mesopores that are considered too small for microorganisms or their extracellular enzymes to reach.^60^ Desorption is considered a prerequisite for subsequent biodegradation,^24,61^ so compounds that adsorb too strongly, with high adsorption energies, may not be available for subsequent biodegradation.^62^ Thus, synergistic effects between adsorption and degradation in GAC filters may be mainly possible for compounds with moderate affinity for adsorption such as diclofenac.

Successive adsorption–desorption was demonstrated for the biologically active compound carbamazepine through the observed differences in breakthrough between the ^14^C-labeled and nonradiolabeled compounds. The retention of compounds with moderate-to-strong adsorption can also have an implication for the evaluation of GAC filters, since the effluent concentrations of micropollutants can be affected by historical variations in the influent, not necessarily captured by conventional sampling campaigns over a few days or a week.

## Supporting Information Available

Figures showing wastewater treatment plant configuration, flow measurements, and ^14^C mass balance calculations; tables showing micropollutant removal in full-scale filters, GAC and sand particle size distribution and column aspect ratios, total and volatile solids, micropollutant concentrations, combustion settings for analysis of solid-phase ^14^C, nonradiolabeled micropollutant LOQ and recovery, and experimental conditions during the initial adaptation period and in reference columns throughout the experiments (PDF)

## References

[ref1] VSA. Liste det ARA mit MV Stufe, die geplant, im Bau oder bereits in Betrieb sind (List of WWTPs with micropollutant removal in operation, planning and under construction in Switzerland). https://micropoll.ch/Mediathek/liste-der-aras-mit-mv-stufe/ (accessed Apr 21, 2024).

[ref2] Svahn O.; Borg S. Assessment of Full-Scale 4th Treatment Step for Micro Pollutant Removal in Sweden: Sand and GAC Filter Combo. Sci. Total Environ. 2024, 906 (October 2023), 167424 10.1016/j.scitotenv.2023.167424. 37793453

[ref3] Takman M.; Svahn O.; Paul C.; Cimbritz M.; Blomqvist S.; Struckmann Poulsen J.; Lund Nielsen J.; Davidsson Å. Assessing the Potential of a Membrane Bioreactor and Granular Activated Carbon Process for Wastewater Reuse – A Full-Scale WWTP Operated over One Year in Scania Sweden. Sci. Total Environ. 2023, 895 (June), 165185 10.1016/j.scitotenv.2023.165185. 37385512

[ref4] Neef J.; Leverenz D.; Launay M. A. Performance of Micropollutant Removal during Wet-Weather Conditions in Advanced Treatment Stages on a Full-Scale WWTP. Water (Switzerland) 2022, 14 ( (20), ). 3281 10.3390/w14203281.

[ref5] Kompetenzzentrum Spurenstoffe Baden-Württemberg. Kläranlagenkarte zur Spurenstoffelimination (Map of wastewater treatment plants with micropollutant removal) https://dwa-bw.maps.arcgis.com/apps/webappviewer/index.html?id=2428880b396f4a21a6123b3fb87feb8b (accessed Apr 21, 2024).

[ref6] Fundneider T.; Acevedo Alonso V.; Abbt-Braun G.; Wick A.; Albrecht D.; Lackner S. Empty Bed Contact Time: The Key for Micropollutant Removal in Activated Carbon Filters. Water Res. 2021, 191, 116765 10.1016/j.watres.2020.116765. 33412419

[ref7] Benstoem F.; Nahrstedt A.; Boehler M.; Knopp G.; Montag D.; Siegrist H.; Pinnekamp J. Performance of Granular Activated Carbon to Remove Micropollutants from Municipal Wastewater—A Meta-Analysis of Pilot- and Large-Scale Studies. Chemosphere. 2017. 185 105 10.1016/j.chemosphere.2017.06.118. 28688844

[ref8] Tchobanoglous G.; Burton F. L.; Stensel H. D. Wastewater Engineering: Treatment and Reuse. Metcalf & Eddy, Inc., 5th ed.; McGraw-Hill Education: New York, 2014; Vol. 1. 10.1016/0309-1708(80)90067-6.

[ref9] Falås P.; Wick A.; Castronovo S.; Habermacher J.; Ternes T. A.; Joss A. Tracing the Limits of Organic Micropollutant Removal in Biological Wastewater Treatment. Water Res. 2016, 95, 240–249. 10.1016/j.watres.2016.03.009. 26999256 PMC5566204

[ref10] Fundneider T.; Acevedo Alonso V.; Wick A.; Albrecht D.; Lackner S. Implications of Biological Activated Carbon Filters for Micropollutant Removal in Wastewater Treatment. Water Res. 2021, 189, 116588 10.1016/j.watres.2020.116588. 33221588

[ref11] Reungoat J.; Escher B. I.; Macova M.; Keller J. Biofiltration of Wastewater Treatment Plant Effluent: Effective Removal of Pharmaceuticals and Personal Care Products and Reduction of Toxicity. Water Res. 2011, 45 (9), 2751–2762. 10.1016/j.watres.2011.02.013. 21450327

[ref12] Altmann J.; Rehfeld D.; Träder K.; Sperlich A.; Jekel M. Combination of Granular Activated Carbon Adsorption and Deep-Bed Filtration as a Single Advanced Wastewater Treatment Step for Organic Micropollutant and Phosphorus Removal. Water Res. 2016, 92, 131–139. 10.1016/j.watres.2016.01.051. 26849316

[ref13] Rattier M.; Reungoat J.; Gernjak W.; Joss A.; Keller J. Investigating the Role of Adsorption and Biodegradation in the Removal of Organic Micropollutants during Biological Activated Carbon Filtration of Treated Wastewater. J. Water Reuse Desalin. 2012, 2 (3), 127–139. 10.2166/wrd.2012.012.

[ref14] Wang H.; Ho L.; Lewis D. M.; Brookes J. D.; Newcombe G. Discriminating and Assessing Adsorption and Biodegradation Removal Mechanisms during Granular Activated Carbon Filtration of Microcystin Toxins. Water Res. 2007, 41 (18), 4262–4270. 10.1016/j.watres.2007.05.057. 17604809

[ref15] Sbardella L.; Comas J.; Fenu A.; Rodriguez-Roda I.; Weemaes M. Advanced Biological Activated Carbon Filter for Removing Pharmaceutically Active Compounds from Treated Wastewater. Sci. Total Environ. 2018, 636, 519–529. 10.1016/j.scitotenv.2018.04.214. 29715656

[ref16] Yuan J.; Mortazavian S.; Crowe G.; Flick R.; Passeport E.; Hofmann R. Evaluating the Relative Adsorption and Biodegradation of 2-Methylisoborneol and Geosmin across Granular Activated Carbon Filter-Adsorbers. Water Res. 2022, 215 (March), 118239 10.1016/j.watres.2022.118239. 35272225

[ref17] Yuan J.; Fox F.; Crowe G.; Mortazavian S.; Passeport E.; Hofmann R. Is In-Service Granular Activated Carbon Biologically Active? An Evaluation of Alternative Experimental Methods to Distinguish Adsorption and Biodegradation in GAC. Environ. Sci. Technol. 2022, 56 (22), 16125–16133. 10.1021/acs.est.2c03639. 36210519

[ref18] Shimabuku K. K.; Zearley T. L.; Dowdell K. S.; Summers R. S. Biodegradation and Attenuation of MIB and 2,4-D in Drinking Water Biologically Active Sand and Activated Carbon Filters. Environ. Sci. Water Res. Technol. 2019, 5 (5), 849–860. 10.1039/C9EW00054B.

[ref19] Sharaf A.; Liu Y. Mechanisms and Kinetics of Greywater Treatment Using Biologically Active Granular Activated Carbon. Chemosphere 2021, 263, 128113 10.1016/j.chemosphere.2020.128113. 33297106

[ref20] Paredes L.; Fernandez-Fontaina E.; Lema J. M.; Omil F.; Carballa M. Understanding the Fate of Organic Micropollutants in Sand and Granular Activated Carbon Bio Fi Ltration Systems. Sci. Total Environ. 2016, 551–552, 640–648. 10.1016/j.scitotenv.2016.02.008. 26897407

[ref21] Yuan J.; Passeport E.; Hofmann R. Understanding Adsorption and Biodegradation in Granular Activated Carbon for Drinking Water Treatment: A Critical Review. Water Res. 2022, 210 (December 2021), 118026 10.1016/j.watres.2021.118026. 34996013

[ref22] Betsholtz A.; Karlsson S.; Svahn O.; Davidsson Å.; Cimbritz M.; Falås P. Tracking 14C-Labeled Organic Micropollutants to Differentiate between Adsorption and Degradation in GAC and Biofilm Processes. Environ. Sci. Technol. 2021, 55 (16), 11318–11327. 10.1021/acs.est.1c02728. 34311545 PMC8383275

[ref23] Speitel G. E.; DiGiano F. A. Bioregeneration of Gac Used To Treat Micropollutants. J./Am. Water Work. Assoc. 1987, 79 (1), 64–73. 10.1002/j.1551-8833.1987.tb02785.x.

[ref24] Speitel G. E.; Lu C. J.; Turakhia M.; Zhu X. J. Biodegradation of Trace Concentrations of Substituted Phenols in Granular Activated Carbon Columns. Environ. Sci. Technol. 1989, 23 (1), 68–74. 10.1021/es00178a008.

[ref25] Putz A. R. H.; Losh D. E.; Speitel G. E. Removal of Nonbiodegradable Chemicals from Mixtures during Granular Activated Carbon Bioregeneration. J. Environ. Eng. 2005, 131 (2), 196–205. 10.1061/(ASCE)0733-9372(2005)131:2(196).

[ref26] Luo Y.; Guo W.; Ngo H. H.; Nghiem L. D.; Hai F. I.; Zhang J.; Liang S.; Wang X. C. A Review on the Occurrence of Micropollutants in the Aquatic Environment and Their Fate and Removal during Wastewater Treatment. Sci. Total Environ. 2014, 473–474, 619–641. 10.1016/j.scitotenv.2013.12.065. 24394371

[ref27] Kovalova L.; Siegrist H.; Von Gunten U.; Eugster J.; Hagenbuch M.; Wittmer A.; Moser R.; McArdell C. S. Elimination of Micropollutants during Post-Treatment of Hospital Wastewater with Powdered Activated Carbon, Ozone, and UV. Environ. Sci. Technol. 2013, 47 (14), 7899–7908. 10.1021/es400708w. 23758546

[ref28] Joss A.; Zabczynski S.; Göbel A.; Hoffmann B.; Löffler D.; McArdell C. S.; Ternes T. A.; Thomsen A.; Siegrist H. Biological Degradation of Pharmaceuticals in Municipal Wastewater Treatment: Proposing a Classification Scheme. Water Res. 2006, 40 (8), 1686–1696. 10.1016/j.watres.2006.02.014. 16620900

[ref29] Margot J.; Kienle C.; Magnet A.; Weil M.; Rossi L.; de Alencastro L. F.; Abegglen C.; Thonney D.; Chèvre N.; Schärer M.; Barry D. A. Treatment of Micropollutants in Municipal Wastewater: Ozone or Powdered Activated Carbon?. Sci. Total Environ. 2013, 461–462, 480–498. 10.1016/j.scitotenv.2013.05.034. 23751332

[ref30] Bourgin M.; Beck B.; Boehler M.; Borowska E.; Fleiner J.; Salhi E.; Teichler R.; von Gunten U.; Siegrist H.; McArdell C. S. Evaluation of a Full-Scale Wastewater Treatment Plant Upgraded with Ozonation and Biological Post-Treatments: Abatement of Micropollutants, Formation of Transformation Products and Oxidation by-Products. Water Res. 2018, 129, 486–498. 10.1016/j.watres.2017.10.036. 29190578

[ref31] Kommineni S.; Ela W. P.; Arnold R. G.; Huling S. G.; Hester B. J.; Betterton E. A. NDMA Treatment by Sequential GAC Adsorption and Fenton-Driven Destruction. Environ. Eng. Sci. 2003, 20 (4), 361–373. 10.1089/109287503322148636.

[ref32] Fleming E. C.; Pennington J. C.; Wachob B. G.; Howe R. A.; Hill D. O. Removal of N-Nitrosodimethylamine from Waters Using Physical-Chemical Techniques. J. Hazard. Mater. 1996, 51 (1–3), 151–164. 10.1016/S0304-3894(96)01833-X.

[ref33] Edefell E.; Svahn O.; Falås P.; Bengtsson E.; Axelsson M.; Ullman R.; Cimbritz M. Digging Deep into a GAC Filter – Temporal and Spatial Profiling of Adsorbed Organic Micropollutants. Water Res. 2022, 218 (October 2021), 118477 10.1016/j.watres.2022.118477. 35487159

[ref34] Sauter D.; Steuer A.; Wasmund K.; Hausmann B.; Szewzyk U.; Sperlich A.; Gnirss R.; Cooper M.; Wintgens T. Microbial Communities and Processes in Biofilters for Post-Treatment of Ozonated Wastewater Treatment Plant Effluent. Sci. Total Environ. 2023, 856 (July 2022), 159265 10.1016/j.scitotenv.2022.159265. 36206900

[ref35] Cimbritz M.; Mattson A. Reningstekniker För Läkemedel Och Mikroföroreningar i Avloppsvatten; 2018.

[ref36] Svahn O.; Björklund E. Describing Sorption of Pharmaceuticals to Lake and River Sediments, and Sewage Sludge from UNESCO Biosphere Reserve Kristianstads Vattenrike by Chromatographic Asymmetry Factors and Recovery Measurements. J. Chromatogr. A 2015, 1415, 73–82. 10.1016/j.chroma.2015.08.061. 26362805

[ref37] Knappe D. R. U.; Snoeyink V. L.; Röche P.; Prados M. J.; Bourbigot M. M. Atrazine Removal by Preloaded GAG. J./Am. Water Work. Assoc. 1999, 91 (10), 97–109. 10.1002/j.1551-8833.1999.tb08719.x.

[ref38] Huang Y.; Nie Z.; Yuan J.; Murray A.; Li Y.; Woods-Chabane G.; Hofmann R. Experimental Validation of a Test to Estimate the Remaining Adsorption Capacity of Granular Activated Carbon for Taste and Odour Compounds. Environ. Sci. Water Res. Technol. 2019, 5 (3), 609–617. 10.1039/C8EW00600H.

[ref39] L’Annunziata M. F.; Kessler M. J. Liquid Scintillation Analysis: Principles and Practice, 3 Ed.; Elsevier, 2013. 10.1016/B978-0-12-384873-4.00007-4.

[ref40] Svahn O.; Björklund E. High Flow-Rate Sample Loading in Large Volume Whole Water Organic Trace Analysis Using Positive Pressure and Finely Ground Sand as a Spe-Column in-Line Filter. Molecules 2019, 24 (7), 1426 10.3390/molecules24071426. 30978956 PMC6479934

[ref41] Svahn O.; Björklund E. Increased Electrospray Ionization Intensities and Expanded Chromatographic Possibilities for Emerging Contaminants Using Mobile Phases of Different PH.. J. Chromatogr. B Anal. Technol. Biomed Life Sci. 2016, 1033–1034, 128–137. 10.1016/j.jchromb.2016.07.015. 27543742

[ref42] Suarez S.; Lema J. M.; Omil F. Removal of Pharmaceutical and Personal Care Products (PPCPs) under Nitrifying and Denitrifying Conditions. Water Res. 2010, 44 (10), 3214–3224. 10.1016/j.watres.2010.02.040. 20338614

[ref43] Langenhoff A.; Inderfurth N.; Veuskens T.; Schraa G.; Blokland M.; Kujawa-Roeleveld K.; Rijnaarts H. Microbial Removal of the Pharmaceutical Compounds Ibuprofen and Diclofenac from Wastewater. Biomed Res. Int. 2013, 2013. 1 10.1155/2013/325806. PMC3852090 24350260

[ref44] Torresi E.; Tang K.; Deng J.; Sund C.; Smets B. F.; Christensson M.; Andersen H. R. Removal of Micropollutants during Biological Phosphorus Removal: Impact of Redox Conditions in MBBR. Sci. Total Environ. 2019, 663, 496–506. 10.1016/j.scitotenv.2019.01.283. 30716641

[ref45] Nalinakumari B.; Cha W.; Fox P. Effects of Primary Substrate Concentration on NDMA Transport during Simulated Aquifer Recharge. J. Environ. Eng. 2010, 136 (4), 363–370. 10.1061/(ASCE)EE.1943-7870.0000168.

[ref46] Bradley P. M.; Carr S. A.; Baird R. B.; Chapelle F. H. Biodegradation of N-Nitrosodimethylamine in Soil from a Water Reclamation Facility. Bioremediat. J. 2005, 9 (2), 115–120. 10.1080/10889860500276607.

[ref47] Jewell K. S.; Falås P.; Wick A.; Joss A.; Ternes T. A. Transformation of Diclofenac in Hybrid Biofilm–Activated Sludge Processes. Water Res. 2016, 105, 559–567. 10.1016/j.watres.2016.08.002. 27690310 PMC5250799

[ref48] Edefell E.; Falås P.; Torresi E.; Hagman M.; Cimbritz M.; Bester K.; Christensson M. Promoting the Degradation of Organic Micropollutants in Tertiary Moving Bed Biofilm Reactors by Controlling Growth and Redox Conditions. J. Hazard. Mater. 2021, 414 (November 2020), 125535 10.1016/j.jhazmat.2021.125535. 33684823

[ref49] Wiese B.; Massmann G.; Jekel M.; Heberer T.; Dünnbier U.; Orlikowski D.; Grützmacher G. Removal Kinetics of Organic Compounds and Sum Parameters under Field Conditions for Managed Aquifer Recharge. Water Res. 2011, 45 (16), 4939–4950. 10.1016/j.watres.2011.06.040. 21835424

[ref50] Tang K.; Ooi G. T. H.; Litty K.; Sundmark K.; Kaarsholm K. M. S.; Sund C.; Kragelund C.; Christensson M.; Bester K.; Andersen H. R. Removal of Pharmaceuticals in Conventionally Treated Wastewater by a Polishing Moving Bed Biofilm Reactor (MBBR) with Intermittent Feeding. Bioresour. Technol. 2017, 236, 77–86. 10.1016/j.biortech.2017.03.159. 28390280

[ref51] Piai L.; Blokland M.; van der Wal A.; Langenhoff A. Biodegradation and Adsorption of Micropollutants by Biological Activated Carbon from a Drinking Water Production Plant. J. Hazard. Mater. 2020, 388 (January), 122028 10.1016/j.jhazmat.2020.122028. 31955023

[ref52] Boehler M.; Hernandez A.; Baggenstos M.; McArdell C. S.; Siegrist H.; Joss A. Elimination von Spurenstoffen Durch Granulierte Aktivkohle-Filtration (GAK): Grosstechnische Untersuchungen Auf Der ARA Furt,Bülach; Duebendorf, Switzwerland, 2020.

[ref53] Zearley T. L.; Summers R. S. Removal of Trace Organic Micropollutants by Drinking Water Biological Filters. Environ. Sci. Technol. 2012, 46 (17), 9412–9419. 10.1021/es301428e. 22881485

[ref54] Tu Y. Y.; Yang C. S. Demethylation and Denitrosation of Nitrosamines by Cytochrome P-450 Isozymes. Arch. Biochem. Biophys. 1985, 242 (1), 32–40. 10.1016/0003-9861(85)90476-X. 4051505

[ref55] Sharp J. O.; Wood T. K.; Alvarez-Cohen L. Aerobic Biodegradation of N-Nitrosodimethylamine (NDMA) by Axenic Bacterial Strains. Biotechnol. Bioeng. 2005, 89 (5), 608–618. 10.1002/bit.20405. 15672376

[ref56] Fournier D.; Hawari J.; Streger S. H.; McClay K.; Hatzinger P. B. Biotransformation of N-Nitrosodimethylamine by Pseudomonas Mendocina KR1. Appl. Environ. Microbiol. 2006, 72 (10), 6693–6698. 10.1128/AEM.01535-06. 16950909 PMC1610310

[ref57] Kaplan D. L.; Kaplan A. M. Biodegradation of N-Nitrosodimethylamine in Aqueous and Soil Systems. Appl. Environ. Microbiol. 1985, 50 (4), 1077–1086. 10.1128/aem.50.4.1077-1086.1985. 16346905 PMC291796

[ref58] Falås P.; Jewell K. S.; Hermes N.; Wick A.; Ternes T. A.; Joss A.; Nielsen J. L. Transformation, CO 2 Formation and Uptake of Four Organic Micropollutants by Carrier-Attached Microorganisms. Water Res. 2018, 141, 405–416. 10.1016/j.watres.2018.03.040. 29859473

[ref59] Svendsen S. B.; El-taliawy H.; Carvalho P. N.; Bester K. Concentration Dependent Degradation of Pharmaceuticals in WWTP Effluent by Biofilm Reactors. Water Research 2020, 186, 116389 10.1016/j.watres.2020.116389. 32916616

[ref60] Aktaş Ö.; Çeçen F. Bioregeneration of Activated Carbon: A Review. Int. Biodeterior. Biodegrad. 2007, 59 (4), 257–272. 10.1016/j.ibiod.2007.01.003.

[ref61] De Jonge R. J.; Breure A. M.; Van Andel J. G. Bioregeneration of Powdered Activated Carbon (PAC) Loaded with Aromatic Compounds. 1996, 30 (4), 875–882. 10.1016/0043-1354(95)00247-2.

[ref62] Orshansky F.; Narkis N. Characteristics of Organics Removal by Pact Simultaneous Adsorption and Biodegradation. Water Res. 1997, 31 (3), 391–398. 10.1016/S0043-1354(96)00227-8.

