# Supplementary material for: New Perspectives on the Interactions between Adsorption and Degradation of Organic Micropollutants in Granular Activated Carbon Filters
Source: Environ Sci Technol. 2024 Jun 18;58(26):11771–80. doi: 10.1021/acs.est.4c00815 (PMC11223462; doi:10.1021/acs.est.4c00815)
Supplement: Supplementary file 1 — es4c00815_si_001.pdf [file es4c00815_si_001.pdf]

Supporting information

New perspectives on the interactions between adsorption and degradation of organic micropollutants in GAC filters

Alexander Betsholtz[†*], Per Falås[†], Ola Svahn[‡], Michael Cimbritz[†], Åsa Davidsson[†]

[†] Department of Process and Life Science Engineering. Division of Chemical Engineering, Lund University, 221 00 Lund, Sweden

[‡] School of Education and Environment, Division of Natural Sciences, Kristianstad University, 291 88 Kristianstad, Sweden

[*]Corresponding authors: email addresses alexander.betsholtz@chemeng.lth.se;

The supporting Information contains
Number of pages: 7
Number of figures: 3
Number of tables: 8

Degeberga WWTP configuration

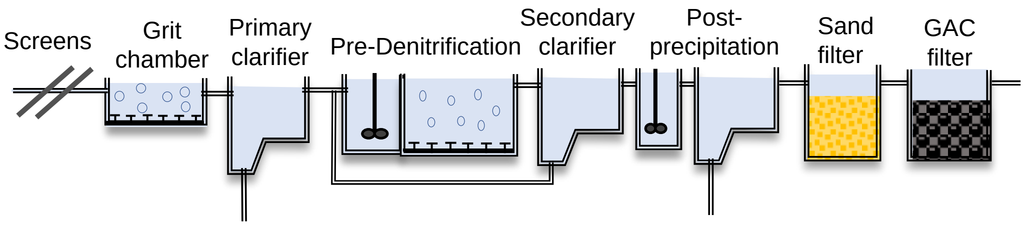

Figure S1. Overview of the configuration of Degeberga WWTP

Micropollutant breakthrough and process conditions in full-scale filters at sample retrieval

The filter influents had high dissolved oxygen concentrations (DO > 8mg/L). Mean EBCTs were 87 min in the sand filter and 45 min in the GAC filter.

Table S1. Number of treated BVs and micropollutant removal (%) for carbamazepine and diclofenac in the full-scale filters at Degeberga WWTP at the time of sample retrieval for each experiment.

**Experiment**	**Sand filter**			**GAC filter**	
	Nr. treated BVs	Carbama. (%)	Diclofenac (%)	Carbama. (%)	Diclofenac (%)
**1**	27,000	5	16	32	87
**2**	25,000	-3	39	45	81
**3**	26,000	5	27	51	91

GAC and sand filter media

Table S2. Particle size distribution, mean particle diameter, and column aspect ratios for the column experiments with GAC and sand.

	**Exp. 1. Anoxic/oxic conditions**		**Exp. 2. Oxic, long EBCT**		**Exp. 3. Oxic, short EBCT**	
**Sieve size [mm]**	GAC (% distr.)	Sand (% distr.)	GAC (% distr.)	Sand (% distr.)	GAC (% distr.)	Sand (% distr.)
1.5–1.4	11.6	0.0	18.4	0.1	14.9	0.1
1.4–1.0	48.7	9.0	49.4	29.5	50.5	30.7
1.0–0.71	32.6	82.3	25.2	65.7	26.9	65
0.7–0.5	6.8	6.0	6.9	2.7	7.4	1.8
< 0.5	0.3	2.8	0.2	1.7	0.3	2.3
**Geometric mean diameter [mm]**						
	1.08	0.87	1.07	0.92	1.04	0.83
**Column aspect ratio [column diameter / particle diameter]**						
	14.8	18.4	15.0	17.4	15.4	19.3

Media weights

Table S3. Dry weights of GAC and sand in the column experiments and volatile solids (VS) content of the sand.

**Experiment**	**Conditions**	**GAC (g/column)***	**Sand (g/column)***	**VS Sand (g VS/kg sand)**
**1**	*Anoxic/oxic conditions*	2.9 ± 0.1	14.6 ± 0.1	23.0 ± 1.7
**2**	*Oxic, long EBCT*	2.9 ± 0.0	15.1 ± 0.2	18.5 ± 0.3
**3**	*Oxic, short EBCT*	3.2 ± 0.1	15.2 ± 0.1	17.6 ± 0.3

*Dry weight

Micropollutant concentrations

Table S4. Initial concentrations of ^{14}C-labeled and corresponding nonradiolabeled compounds in the experimental feeds.

	**Added ^{14}C-MP concentration (ng/L)**	**Added nonradiolabeled MP concentration (ng/L)**	**Measured nonradiolabeled MP concentrations***
**Carbamazepine**	1050	1050	3700 ± 300
**Diclofenac**	530	530	1400 ± 100
**NDMA**	135	135	**

*Sum of spiked and background concentrations.

**Not measured.

Combustion settings

Table S5. Settings for the combustion program for the analysis of solid-phase ^{14}C.

Setting	Heating (°C/min)	Time (min)	Temperature (°C)	Gasflow	Catalyst zone temperature (°C)
**Rate**	3	~60	to 200	Air	900
**Dwell**	-	30	200	Air	900
**Rate**	3	~30	to 300	O_2	900
**Dwell**	-	20	300	O_2	900
**Rate**	5	60	to 600	Air	900
**Dwell**	-	20	600	Air	900
**Rate**	10	30	to 900	O_2	900
**Dwell**	-	30	900	O_2	900

Micropollutant analysis

Table S6. Limit of quantification (LOQ), relative standard deviation (RSD) of the method, and recovery of internal standards for the analysis of nonradiolabeled micropollutants.

	LOQ (ng/L)	RSD (%)	Internal standard	Recovery (%)
**Carbamazepine**	0.5	0.7	Carbamazepine-d10	96
**Diclofenac**	1.0	3.0	Diclofenac-C6	97

Experimental conditions

Table S7. Experimental conditions during the initial adaptation period**.**

Experiment 1: Anoxic/oxic conditions			
**Parameter**	**Feed (n=3)**	**Out GAC filter (n=4)**	**Out Sand filter (n=4)**
**DO [mg/L]**	< 0.1	-	-
**pH [-]**	6.7–7.4	-	-
**T [°C]**	19–22	-	-
**DOC [mgC/L]**	5.9 ± 0.3	5.7 ± 0.1	5.8 ± 0.2
**UVA_{254} [m^{-1}]**	12.9 ± 0.4	13.2 ± 0.5	12.8 ± 0.5
**NH_4^+-N [mg/L]**	0.7 ± 0.0	0.7 ± 0.0	0.7 ± 0.0
**NO_3^--N [mg/L]**	9.5 ± 0.0	9.3 ± 0.1	9.4 ± 0.1
**NO_2^--N [mg/L]**	< 0.1	0.2 ± 0.0	0.1 ± 0.0
Experiment 2: Oxic, long EBCT			
**Parameter**	**Feed (n=3)**	**Out GAC filter (n=4)**	**Out Sand filter (n=4)**
**DO [mg/L]**	> 8.0	-	-
**pH [-]**	6.9–7.8	-	-
**T [°C]**	19–21	-	-
**DOC [mgC/L]**	5.6 ± 0.1	5.3 ± 0.1	5.3 ± 0.2
**UVA_{254} [m^{-1}]**	15 ± 0.0	13.6 ± 0.1	14.4 ± 0.2
**NH_4^+-N [mg/L]**	< 0.1–0.2	< 0.1	< 0.1
**NO_3^--N [mg/L]**	13.5 ± 0.0	13.5 ± 0.1	13.5 ± 0.1
**NO_2^--N [mg/L]**	< 0.1	< 0.1	< 0.1
Experiment 3: Oxic, short EBCT			
**Parameter**	**Feed (n=3)**	**Out GAC filter (n=4)**	**Out Sand filter (n=4)**
**DO [mg/L]**	> 8.0	-	-
**pH [-]**	7.0–7.5	-	-
**T [°C]**	20–21	-	-
**DOC [mgC/L]**	5.6 ± 0.1	5.3 ± 0.1	5.4 ± 0.1
**UVA_{254} [m^{-1}]**	13.8 ± 0.0	13.4 ± 0.1	13.7 ± 0.0
**NH_4^+-N [mg/L]**	< 0.1	< 0.1	< 0.1
**NO_3^--N [mg/L]**	17.3 ± 0.0	17.3 ± 0.0	17.3 ± 0.1
**NO_2^--N [mg/L]**	< 0.1	< 0.1	< 0.1

Table S8. Experimental conditions for measurements in reference columns with nonradiolabeled micropollutants.

	Experiment 1a: Anoxic conditions		
**Parameter**	**Feed (n=3)**	**Out GAC filter (n=6)**	**Out Sand filter (n=6)**
**DO [mg/L]**	< 0.1	-	-
**pH [-]**	6.8–7.8	-	-
**T [°C]**	20–23	-	-
**DOC [mgC/L]**	5.9 ± 0.3	5.4 ± 0.1	5.3 ± 0.2
**UVA_{254} [m^{-1}]**	12.9 ± 0.4	11.8 ± 0.7	11.3 ± 0.3
**NH_4^+-N [mg/L]**	0.7 ± 0.0	0.7 ± 0.0	0.7 ± 0.0
**NO_3^--N [mg/L]**	9.5 ± 0.0	9.2 ± 0.0	9.3 ± 0.0
**NO_2^--N [mg/L]**	< 0.1	< 0.1	< 0.1
	Experiment 1b: Oxic conditions		
**Parameter**	**Feed (n=3)**	**Out GAC filter (n=6)**	**Out Sand filter (n=6)**
**DO [mg/L]**	> 8	-	-
**pH [-]**	6.8–7.8	-	-
**T [°C]**	20–23	-	-
**DOC [mgC/L]**	5.9 ± 0.3	5.1 ± 0.2	4.9 ± 0.2
**UVA_{254} [m^{-1}]**	12.9 ± 0.4	10.9 ± 0.4	10.9 ± 0.3
**NH_4^+-N [mg/L]**	0.7 ± 0.0	<0.1 – 0.4	<0.1 – 0.3
**NO_3^--N [mg/L]**	9.5 ± 0.0	10.0 ± 0.2	10.1 ± 0.1
**NO_2^--N [mg/L]**	< 0.1	< 0.1	< 0.1
	Experiment 2: Oxic, long EBCT		
**Parameter**	**Feed (n=3)**	**Out GAC filter (n=6)**	**Out Sand filter (n=6)**
**DO [mg/L]**	> 8.0	-	-
**pH [-]**	6.9–7.8	-	-
**T [°C]**	19–21	-	-
**DOC [mgC/L]**	5.6 ± 0.1	5.2 ± 0.2	5.3 ± 0.2
**UVA_{254} [m^{-1}]**	15 ± 0.0	12.9 ± 0.6	13.7 ± 0.5
**NH_4^+-N [mg/L]**	< 0.1–0.2	-	-
**NO_3^--N [mg/L]**	13.5 ± 0.0	-	-
**NO_2^--N [mg/L]**	< 0.1	-	-
	Experiment 3: Oxic, short EBCT		
**Parameter**	**Feed (n=3)**	**Out GAC filter (n=5)**	**Out Sand filter (n=5)**
**DO [mg/L]**	> 8.0	-	-
**pH [-]**	7.0–7.6	-	-
**T [°C]**	19–21	-	-
**DOC [mgC/L]**	5.6 ± 0.1	5.1 ± 0.1	5.3 ± 0.1
**UVA_{254} [m^{-1}]**	13.8 ± 0.0	12.1 ± 0.3	12.9 ± 0.1
**NH_4^+-N [mg/L]**	< 0.1	-	-
**NO_3^--N [mg/L]**	17.3 ± 0.0	-	-
**NO_2^--N [mg/L]**	< 0.1	-	-

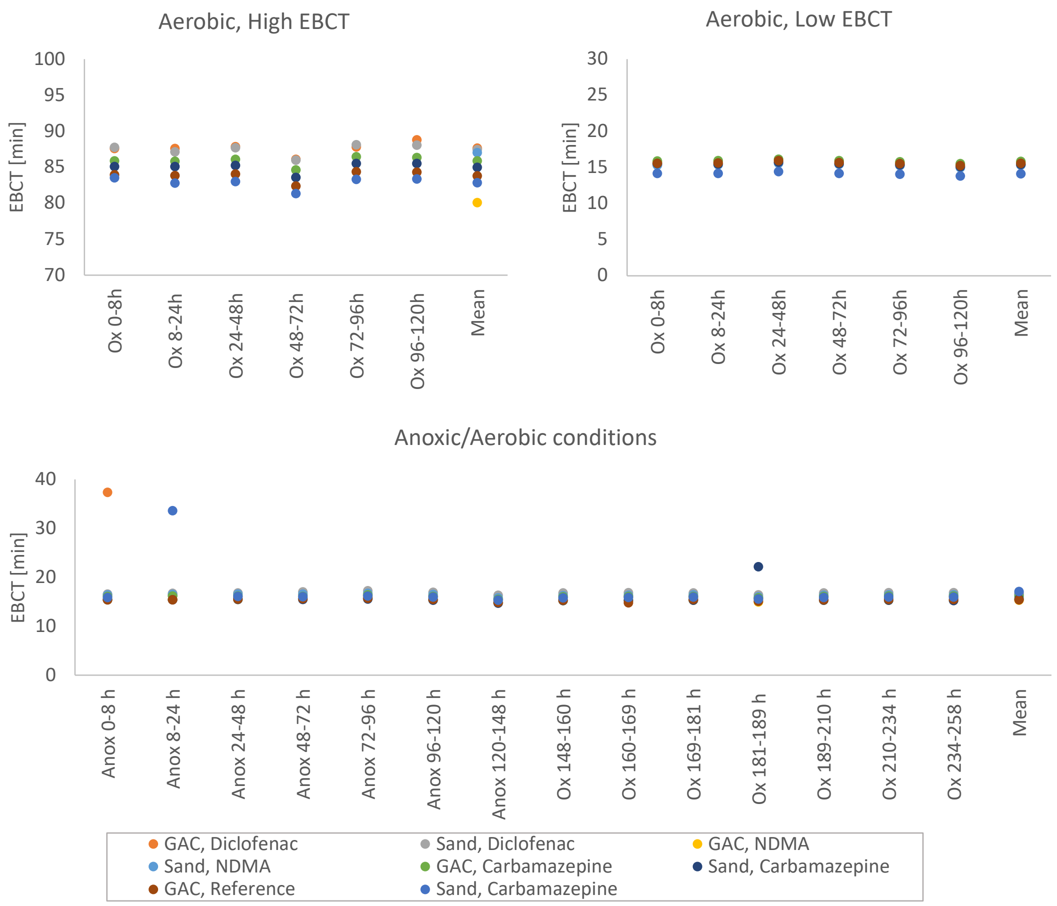

Figure S2. Measurements of flow (EBCTs) during the column experiments.

^{14}C mass balance calculations

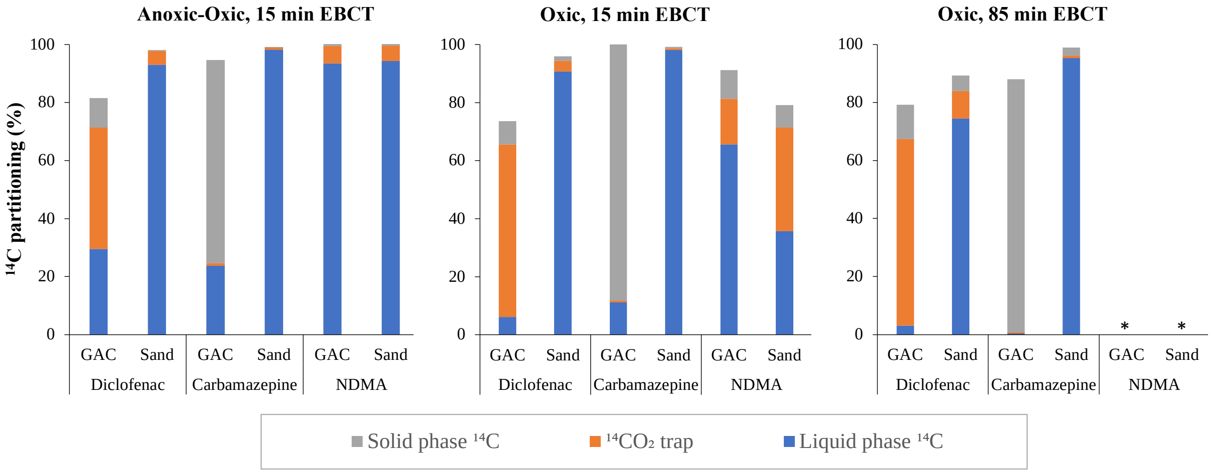

Figure S3. Partitioning of ^{14}C activity between solid phase (adsorbed), CO_2 trap, and liquid phase at the end of the experiment. The values for ^{14}C in the liquid phase and CO_2 trap are accumulated fractions during the entire experiment. *No data available due to experimental error.
